# Political partisanship and perceived partisan threat relate to simple trust decisions

**DOI:** 10.1186/s41235-025-00698-3

**Published:** 2026-01-03

**Authors:** Brittany S. Cassidy, Junaid Rasool, Israel W. Smitherman, BoKyung Park, Kendra L. Seaman

**Affiliations:** 1 Department of Psychology, University of North Carolin at Greensboro, 296 Eberhart, PO Box 26180, Greensboro, NC 27412 USA; 2 University of Texas at Dallas, Dallas, Texas USA; 3 Center for Vital Longevity, University of Texas at Dallas, Dallas, Texas USA

**Keywords:** Political partisanship, Threat, Trust, Economic decision making

## Abstract

Affective polarization in the USA is an ongoing and pervasive problem eroding cooperation and productive debate related to partisan and non-partisan topics. Such findings signal an inherent mistrust of opposing partisans that is likely related to the extent of threat people believe opposing partisans pose to them. Whereas past work has examined the mistrust and threat characteristic of affective polarization in complex social environments as well as in social perception, no work to date has examined their effects in the context of simple everyday decisions. The current work filled this gap in the literature by examining effects of political partisanship and perceived partisan threat on trust behavior indexed by a trust game widely used in decision-making research. Across two studies using college-aged adults (Study 1) and adults across the lifespan (Study 2), participant and target partisanship interacted to affect trust. Across studies, more liberal participants exhibited lower trust toward Republicans than Democrats. However, more conservative participants’ trust either remained stable across party lines (Study 1) or exhibited lower trust toward Democrats than Republicans (Study 2). In Study 1, these patterns were paralleled when substituting perceived partisan threat toward Republicans and Democrats for political ideology. Study 2 manipulated partisan threat to show larger participant-based partisan differences in trust when partisan partners were more versus less threatening. These findings in part support threat-related affective polarization in trust decisions and have implications for intergroup interactions along partisan lines in everyday life.

**Supplementary Information:**

The online version contains supplementary material available at 10.1186/s41235-025-00698-3.

## Significance

Political polarization poses a pervasive problem both in the USA and worldwide because it produces animosity undermining bipartisan cooperation. This animosity extends from discourse surrounding policy to relatively automatic social inferences (e.g., the first impressions people derive from facial appearance). An open question regards whether political partisanship also affects simple behavioral decisions where both perceivers and targets may benefit from the extension of trust across party lines. This question is important to consider because partisans interact in everyday life in contexts in which their partisanship is irrelevant to the tasks at hand. To fill this gap in the literature, we used a well-established decision-making paradigm to examine how target and perceiver partisanship interact to affect behavioral trust decisions. Here, more liberal participants exhibited lower trust behavior in Republicans than Democrats. By contrast, more conservative participants’ trust behavior either remained relatively stable across party lines or erred toward lower trust toward Democrats than Republicans. These patterns were largely paralleled when relating perceived threat from Democrats and Republicans to trust behavior toward partisans. Perceived partisan threat, moreover, plays a causal role in predicting trust behavior toward partisans. These findings add to the literature by showing that threat-based responses contribute to partisans’ social behavior even in decision-making tasks when partisanship is irrelevant.

Political polarization is an ongoing topic of worldwide social science research (Finkel et al., 2020; Iyengar & Westwood, 2015; Iyengar et al., 2019). This research once focused on interrogating the possibility that polarization stems from differences in policy-related beliefs (e.g., Abramowitz & Saunders, 2008). More recent research has focused on affective polarization, or the division reflected by increasing animosity toward opposing partisans (Iyengar et al., 2019). Such increases positively relate to party loyalty even among people who are partisan yet independent-leaning (Abramowitz & Webster, 2016). Affective polarization is corrosive to complex social interactions in partisan and non-partisan contexts where people have the opportunity to engage in debate (Mamakos & Finkel, 2023). It is also evident in social perception. Indeed, people evaluate the faces of opposing versus similar partisans as less attractive (Mallinas et al., 2018), competent (Cassidy et al., 2022), and trustworthy (Malloy et al., 2023). In both cases, such patterns relate to a broader idea that opposing partisans are threatening and thus not to be trusted (Renström et al., 2021). These findings suggest that affective polarization is likely present in everyday social decisions in which partisanship is irrelevant. Addressing this possibility is important to fully characterize if and how affective, threat-driven polarization shapes our social landscape and the potential for bridging divides among people belonging to different political coalitions (Abramowitz & Webster, 2016). To fill this gap in the literature, the current research was designed to assess effects of political partisanship and perceived partisan threat on behavioral trust decisions.

One way researchers have examined trust decisions is through evaluating behavior in a trust game (Berg et al., 1995). In this game, participants are endowed with points and can share these points with partners in increments. Points shared with partners are then multiplied. The partner then has the option of sharing half the proceeds or keeping all the points. In the latter outcome, participants receive nothing. Whereas cooperation and trust lead to gains for both participants and partners, noncooperation and mistrust leads to losses for participants. Here, the number of points shared with partners is a behavioral indicator of trust (e.g., Seaman et al., 2023). The composition of trust games varies. Some tasks are one-shot and provide an index of trust behavior through a single interaction with each partner. Others are iterative in nature and involve repeated interactions with partners to characterize trust as a learned social behavior (King-Casas et al., 2005).

To characterize trust behavior, the current research used a trust game. Here, partners were labeled with an American political party (i.e., Republican or Democrat). Thus, trust behavior could be assessed via combinations of partner political party and participant political ideology. We first used participant political ideology as a measure of perceiver partisanship for several reasons. First, it is a single continuous variable relating to partisan prejudice (Brandt, 2017) that may better capture a range of beliefs than a binary categorical distinction. Second, and relatedly, people have biased perceptions of their political orientations (Zell & Bernstein, 2014) and may categorize themselves in ways that do not reflect their true beliefs. By using a continuous measure of ideology, we may better capture nuances in perception. Third, even though ideology (ranging from conservative to liberal) does not exactly match Republican and Democrat labels, these correlated concepts have been used in past research examining partisanship effects on social perception (Wilson & Rule, 2014). Here, we expected that with political ideologies shifting among participants from being more ideologically conservative to more liberal, participants would trust Democrat partners more, but Republican partners less.

Finding that people make trust decisions favoring similar versus opposing partisans would be expected by the broader literature on intergroup interactions (Tajfel, 1970). Indeed, classic work on social identity asserts that mere categorization elicits intergroup differences in behavior that favors ingroup over outgroup members (for a review, see Hogg, 2016). Yet, these differences can have different motivations. People can be motivated by “ingroup love” and “outgroup hate,” which are often considered separable constructs (Weisel & Böhm, 2015). Further, these constructs are not symmetric in practice, with much work supporting that intergroup discrimination is primarily motivated by favoring ingroup members (rather than derogating outgroup members; Brewer, 1999).

Contrasting this literature, work on affective polarization suggests that intergroup differences reflect political sectarianism characterized by othering and aversion to opposing partisans (Finkel et al., 2020). Threat from an ideological outgroup has been identified as a potential mechanism for affective polarization. For example, gaps in perceived threat between competing ideological groups relate to higher levels of affective polarization in both South Korea and Israel (Harel et al., 2024). Similar patterns have been observed in work on the trust perceived in faces (e.g., Cassidy et al., 2022). Suggesting that perceived threat plays a causal role in partisan trust, experimentally manipulating inter-party threat strengthens opposing partisan derogation in face impressions (Malloy et al., 2023). These findings show that threat is essential to consider when examining affective polarization among partisans (Renström et al., 2021). To date, however, no research has assessed threat perception in the context of a trust game. To address this gap in the literature and to further support threat as a mechanism for behavior between partisans, we examined whether the above-described interactive pattern of participant political ideology and partner political party on trust behavior would be paralleled by a similar interactive pattern between participant ideology and perceived partisan threat. Based on these findings, we expected the above-described interactive pattern would be paralleled when examining perceived partisan threat instead of participant ideology. Here, we expected that participants would share more with partners belonging to political parties they viewed as less threatening.

The current work provides a conceptual replication of work on trust in first impressions in faces (Cassidy et al., 2022; Malloy et al., 2023) that also extends it by using an established paradigm widely used in the decision-making literature (Berg et al., 1995; King-Casas et al., 2005; Seaman et al., 2023). Beyond the theoretical benefit of demonstrating partisanship effects on behavior that is often the *consequence* of face impressions (Todorov, 2008; Todorov et al., 2014), the current work goes beyond a conceptual replication by also providing analyses that potentially forge a link between participant ideology, perceived partisan threat, and trust behavior. We provide correlational (Study 1) and experimental (Study 2) evidence that perceived threat is vital to interpreting relations between participant political ideology and trust behavior toward partisans.

### Study 1

Study 1 was a test of whether people’s political ideology relates to how they make simple trust decisions involving partisans. We expected that with political ideologies shifting among participants from being more ideologically conservative to more liberal, participants would trust Democrat partners more, but Republican partners less. Because impressions of partisans relate to perceived partisan threat (e.g., Malloy et al., 2023), we expected these relations to emerge when substituting participants’ perceived partisan threat for ideology. These hypotheses were preregistered prior to data analysis (https://osf.io/3dr7k).

## Method

### Participants

114 participants (18–35 years old) who could read and understand English were recruited from two universities in the southern USA (63 from the University of North Carolina at Greensboro and 51 from the University of Texas at Dallas) and their surrounding communities. Across experiments, all participants provided informed consent. All experiments and procedures were approved by the University of North Carolina at Greensboro and University of Texas at Dallas Institutional Review Boards.

## Procedure

Trust was quantified in a game commonly used to assess decision making (Berg et al., 1995). In the game, participants were endowed with four points at the beginning of each trial. In each trial, participants chose whether to share none (0), some (1–3), or all (4) of their points with a partner. These shared points were quadrupled (i.e., sharing two points resulted in the partner now having eight points). Following the choice, the partner decided whether to share half of these points back with the participant or keep all the points for themselves. See below for a description of the feedback procedure. Partners were represented by a picture of a neutrally expressive White male face, a name, and political party (Democrat or Republican) on screen. Partner faces, names, and political parties were randomized for each participant. Participants made decisions about different partners in a random order.

### Limiting analyses to first encounter with each unique partner

The analyses included in this paper were part of a larger preregistered project examining differences both in first trust decisions *and* trust decisions after repeated encounters. After data collection, however, it became clear that a programming error caused the task to include 45 total trials (instead of 60). Participants interacted with each of the six unique partners an average of 7.5 times (range: 1–13 time) over the course of the task. This error led to an unbalanced design rendering the analyses of trust decisions after repeated encounters untenable. Based on this issue, we revised our preregistration prior to data analyses to focus on the first trust decision participants made with each partner. Both the original and revised preregistrations can be viewed on the Open Science Framework by toggling between the “Latest” and “Original” menu in the upper left-hand corner of the preregistration (https://osf.io/wmf4b?revisionId=65e0fa00bce8e502a7855a07).

In the service of our first hypothesis, we limited analyses to the first trial with each unique partner to isolate, in part, effects prior to any learning based on feedback (i.e., the amount shared back to participants from partners). We thus analyzed six trials per participant, which corresponded with the first encounter with each of the six unique partners. Although conducting analyses in this way is consistent with our preregistered hypothesis, one concern is that these six trials were more than likely not the first six trials completed in the experiment. In the results section, we briefly describe analyses (for details, see Supplemental Material) designed to address this concern.

### Feedback procedure

After participants’ trust choices, partners either shared half of the points back with the participants or kept all the points for themselves. This procedure is consistent with repeated trust games (e.g., King-Casas et al., 2005). For completeness, we provide details on the feedback procedure we originally planned to use here. We planned to systematically vary feedback to make partners appear relatively trustworthy, neutral, or untrustworthy (e.g., FeldmanHall et al., 2018). Of the three Republican [Democrat] partners, one was planned to be trustworthy, one neutral, and one untrustworthy. Trustworthy partners shared on 93% of trials. Neutral partners shared on 60% of trials. Untrustworthy partners shared on 7% of trials. Unfortunately, due to programming errors, these sharing rates were not achieved, so this aspect of the original project was abandoned.

### Participant characterization

Participants indicated their overall political ideology as well as their ideologies on social, economic, and foreign policy issues on scales ranging from 1 (*extremely conservative*) to 7 (*extremely liberal*) over four items as used in related work (Cassidy et al., 2022; Malloy et al., 2023). Composite ideology scores were created by averaging responses to the items (Cronbach’s *α* = .86).

Participants next indicated their perceived threat from Republicans, Democrats, and Independent/undecided individuals on scales ranging from 1 (*not at all*) to 7 (*very much*). For each group, participants indicated how much threat they believed [a person/an elected official] posed to society and to themselves. Responses to Republicans (Cronbach’s *α* = .89), Democrats (Cronbach’s *α* = .86), and Independent/undecided individuals (Cronbach’s *α* = .84) were averaged to create three composite partisan threat scores. See Table 1 for descriptives and intercorrelations.

**Table 1:** Descriptives and intercorrelations with confidence intervals for participant characterization variables in Study 1 (below the diagonal) and Study 2 (above the diagonal)

Variable	*Study 1 M (SD)*	*Study 2 M (SD)*	1	2	3	4
1. Composite Ideology	4.75 (1.12)	4.62 (1.62)		.55**	-.36**	.13
				[.45, .64]	[-.47, -.23]	[-.01, .26]
2. Composite Republican Threat	4.23 (1.63)	4.09 (1.92)	.48**		-.28**	.26**
			[.42, .54]		[-.40, -.14]	[.12, .38]
3. Composite Democrat Threat	3.10 (1.28)	3.35 (1.79)	-.04	.45**		.51**
			[-.12, .04]	[.38, .51]		[.39, .60]
4. Composite Independent Threat	2.33 (1.15)	3.19 (1.45)	.21**	.32**	.53**	
			[.13, .29]	[.24, .38]	[.47, .58]	

*Note. M* and *SD* are used to represent mean and standard deviation, respectively. Values in square brackets indicate the 95% confidence interval for each correlation. The confidence interval is a plausible range of population correlations that could have caused the sample correlation. ** indicates *p* < .01

### Statistical transparency

Across experiments, all data cleaning, analyses, and plotting were done using R. All analytic code is available at https://osf.io/5b4gs/?view_only=b6a9c9b13e734288be8c946b6fcc947b. We used *lme4* to run multilevel models and *lmerTest* for p-value calculations. The linear models in the exploratory analyses were conducted using base R. *lsr* was used for producing effect sizes for one-sample t-tests. We acknowledge that directional information regarding contrasts driving interaction effects can be obtained from model coefficients. However, we include, where relevant, descriptive slope information about interactions across studies for three reasons. First, including this information is consistent with psychological research conventions (e.g., Aguinis & Gottfredson, 2010.; Jie et al., 2015; but see Garofalo et al., 2022). Second, they provide within-text descriptive information so readers can explicitly discern the extent to which the data aligned with our hypotheses. Third, they can explicitly clarify the directional nature of effects to the reader. We obtained estimated marginal means and contrasts to describe interaction effects using *emmeans*. We used *emtrends* to obtain simple slope information to describe interaction effects.

## Results

### Relations with target political partisanship and perceiver ideology

We first tested the preregistered hypothesis that with political ideologies shifting among participants from more conservative to liberal, participants would trust Democrat partners more and Republican partners less. In a multilevel model, we regressed trust (standardized) on Partner Political Party (Republican or Democrat), Participant Political Ideology (standardized composite political ideology scores), and their interaction. Here, trust is defined as the first amount participants shared with each unique partner. The random effects structure allowed for intercepts to vary by participant. It also included a random slope such that the Partner Political Party effect was allowed to vary by participants. See Table 2 for model coefficient information beyond the below-described effects. Across studies, we standardized the dependent variable for analyses. Presented coefficients are thus standardized betas, which allows for a direct comparison of predictor strength.

**Table 2:** Effects of participant ideology and partner political party on the amount of the first trust decision with each unique partner in Study 1. Model coefficients are standardized

	Amount of first trust decision (standardized)		
*Predictors*	*Estimates*	*CI*	*p*
(Intercept)	0.18	0.02–0.33	0.024
Participant ideology (standardized)	0.01	− 0.15–0.18	0.858
Partner political party [Republican]	− 0.35	− 0.51–− 0.19	< 0.001
Participant ideology × Partner political party [Republican]	− 0.26	− 0.43–− 0.08	0.004

**Fig. 1:**
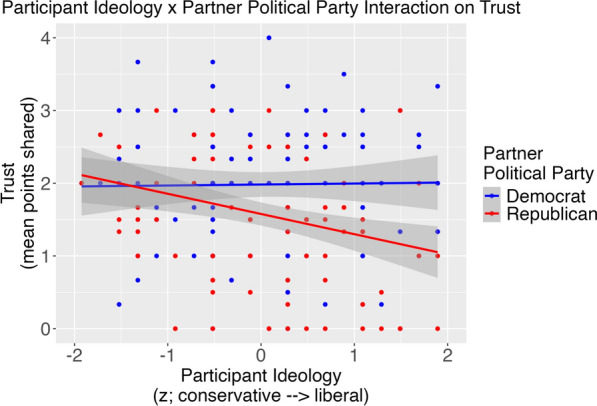
An interaction effect obtained from a multilevel model showed that participant political ideology (conservative → liberal) negatively related to trust in Republican, but not Democrat, partners in Study 1

A Partner Political Party effect emerged, *B* = − 0.35, *p* < .001, such that participants shared more to Democrat versus Republican partners. The Partner Political Party effect was qualified by Participant Political Ideology in a way that partially supported our preregistered hypothesis, *B* = − 0.26, *p* = .004 (Fig. 1). For descriptive purposes, we obtained simple slopes. Supporting our hypothesis, participants with more liberal political ideologies trusted Republican partners less, *B* = − 0.25, *SE* = 0.08. Contrary to our hypothesis, the relation between Participant Political Ideology and trust toward Democrat partners was relatively flat, *B* = 0.01, *SE* = 0.09.

### Potential feedback exposure concerns

A potential concern regarding the above-described results is that the analyzed trials were likely not the first six completed in the experiment. This choice may limit, in part, exposure to partner feedback, leading to learning that could influence subsequent trust decisions (see FeldmanHall et al., 2018; Seaman et al., 2023). However, limiting exposure does not eliminate it, meaning that the presented results may not reflect “pure” trust decisions. We conducted an additional analysis designed to address these potential concerns. Here, we isolated the first encounter with the *second* Democrat and Republican partners encountered. We also determined the earnings from the trial with the *first* Democrat and Republican partners encountered, which reflects the extent to which feedback from Republicans or Democrats benefited the participant. In a multilevel model, we regressed trust (standardized) on Partner Political Party (Republican or Democrat), Participant Political Ideology (standardized composite political ideology scores), Last Trial Earnings (standardized around the overall mean), and their interactions. There was a positive effect of Last Trial Earnings, *B* = 0.20, *p* = .004. As would be expected, the more participants received in their first encounter with a Republican or Democrat partner, the more they shared in an encounter with a partner of the same party. Critically, the interaction between Participant Political Ideology and Partner Political Party remained significant, *B* = − 0.16, *p* = .023. Simple slopes reflecting this interaction replicated the simple slope patterns in the above-described analysis. See the Supplemental Material for complete information about this model.

### Relations with target political partisanship and perceived partisan threat

**Fig. 2:**
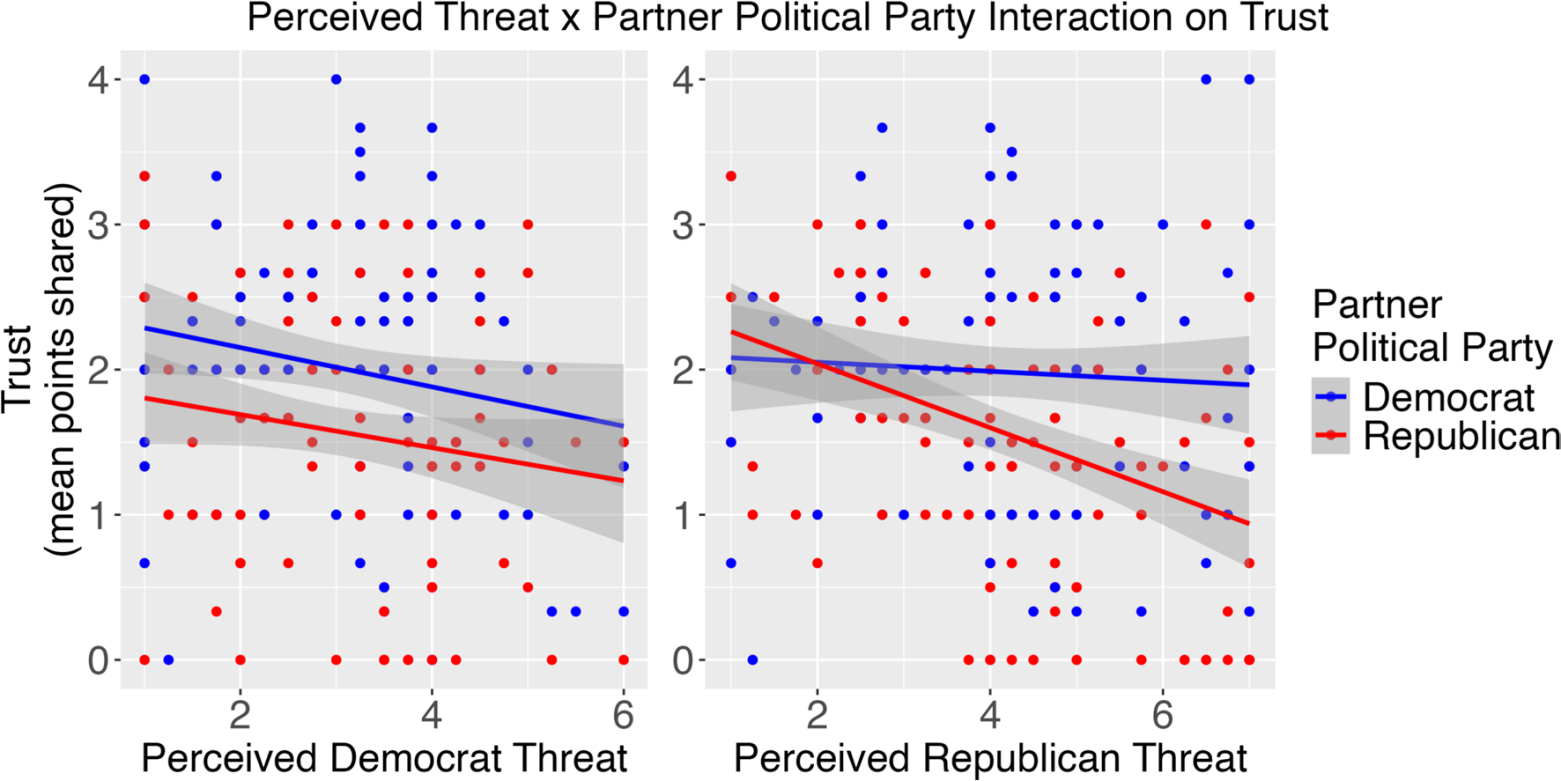
An interaction effect obtained from a multilevel model showed that partisan threat (less → more) only negatively related to trust toward partners belonging to the corresponding party in Study 1

We next tested the hypothesis that the above-described patterns involving participant political ideology would be paralleled by patterns involving perceived partisan threat. Here, we restrict our depiction to effects that paralleled this interactive pattern. In a multilevel model, we regressed trust (i.e., the points shared in the first trial of the trust game; standardized) on Partner Political Party (Republican or Democrat), perceived threat toward Democrats, Republicans and Independents/Undecided (each standardized around the overall composite threat scores for the respective party), and three two-way interaction terms (Partner Political Party x Perceived [Democrat/Republican/Independent] Threat). The random effect structure allowed for intercepts to vary by participant. It also included a random slope allowing partner political party effects to vary by participant. See Table 3 for model coefficient information beyond the below-described significant effects. Only threat from Republicans and Democrats qualified Partner Political Party effects (Fig. 2). To describe how our hypotheses were supported, we obtained simple slopes.

**Table 3:** Effects of perceived partisan threat and partner political party on the amount of the first trust decision with each unique partner in Study 1. Model coefficients are standardized

	Amount of first trust decision (standardized)		
*Predictors*	*Estimates*	*CI*	*p*
(Intercept)	0.18	0.03–0.33	0.022
Democrat threat (standardized)	− 0.25	− 0.43–− 0.06	0.011
Partner political party [Republican]	− 0.37	− 0.53–− 0.22	< 0.001
Republican threat (standardized)	0.05	− 0.13–0.22	0.589
Independent threat (standardized)	0.03	− 0.15–0.21	0.781
Democrat threat × Partner political party [Republican]	0.21	0.02–0.40	0.035
Republican threat × Partner political party [Republican]	− 0.39	− 0.57–− 0.21	< 0.001
Independent threat × Partner political party [Republican]	0.08	− 0.11–0.26	0.412

### Perceived threat from Democrats

We first used simple slopes to describe the interaction between threat from Democrats and Partner Political party on trust, *B* = 0.21, *p* = .04. As threat from Democrats increased, trust toward Democrat partners decreased *B* = − 0.25, *SE* = 0.10. By contrast, the relation between Perceived Democrat Threat and trust toward Republican partners was relatively flat, *B* = − 0.03, *SE* = 0.09.

### Perceived threat from Republicans

We then examined the interaction between threat from Republicans and Partner Political Party on trust, *B* = − 0.39, *p* < .001, in the same manner as we did for threat from Democrats. As threat from Republicans increased, trust toward Republican partners decreased, *B* = − 0.34, *SE* = 0.08. By contrast, the relation between Perceived Republican threat and trust toward Democrat partners was relatively flat, *B* = 0.05, *SE* = 0.09.

## Discussion

We expected that having a relatively more liberal ideology would positively relate to trust (i.e., sharing more points) with Democrat partners. By contrast, we expected a negative relation with having a more liberal ideology and trust placed in Republican partners. The data partially supported these predictions. Supporting our predictions, participant political ideology negatively related to trust placed in Republican partners. It did not significantly relate to trust placed in Democrat partners. This pattern means that whereas more liberal versus conservative participants trusted Republican partners less, they similarly trusted Democrat partners.

That higher liberalism negatively related to trust in Republicans and that more liberal versus conservative participants trusted Republican partners less reflects work on affective polarization (Iyengar et al., 2019). These patterns reflect that, at least among more liberal participants, the increasing prominence of outgroup derogation in cross-partisan interactions reflective of political sectarianism (Finkel et al., 2020). Yet, similar trust toward Democrat partners from relatively more liberal versus conservative participants does not align with this interpretation. What might explain this pattern?

One explanation may lie within the ideological composition of our participant sample. Note that a one-sample t-test against the ideology scale midpoint showed that the mean political ideology of the sample was shifted toward the ideologically liberal end of the scale. Thus, examining relatively more conservative participants may reflect more moderate ideological views than the more extreme views associated with opposing partisan derogation (Iyengar & Westwood, 2015). Past work has also illustrated asymmetric patterns. For example, disclosing the partisanship of target faces affected face impressions of more liberal, but not more conservative participants in a sample of college-students (Cassidy et al., 2022). Second, the extent to which liberals derogate conservatives is linked to a moralized belief that counter-partisans pose harm to disadvantaged groups (Nair et al., 2025). Thus, interactive partisanship effects might reflect a unique form of threat that is more likely to drive partisan animus from Democrats than Republicans. Future work may assess these possibilities.

More general threat perceptions showed that threat contributes to how people make trust decisions toward partisans. Participants shared less with partners belonging to political parties they viewed as more threatening. These findings parallel work showing correlational (Cassidy et al., 2022) and experimental (Malloy et al., 2023) evidence that perceived threat is essential to examining behavior likely reflecting affective polarization (Renström et al., 2021). That the perceived threat from independents/undecideds did not affect trust behavior suggests that it is the affect-laden oppositional group, and not group status overall, that relates to trust behavior. These patterns also suggest that *specific* partisan threat may signal trust for *specific* partisans. Indeed, when people perceive more (versus less) Republican threat, they only trust Republican partners less. Likewise, when people perceive more (versus less) Democrat threat, they only trust Democrat partners less.

That complementary patterns emerged on trust behavior with partisan threat, and not ideology, suggest threat to be an essential component to cross-partisan behavior. One possibility is that perceived threat constrains the extent to which participant ideology relates to trust behavior toward partisans. Because the analyses in Study 1 were correlational, we cannot draw causal inferences from them to supporting this possibility (see Fiedler et al., 2011). To this end, we designed Study 2 to test effects of an experimental threat manipulation on interactive partisanship effects on trust behavior.

### Study 2

Study 2 extended Study 1 by including an experimental manipulation of partisan threat. By experimentally manipulating the amount of partisan threat, we could test whether the extent of partisan threat moderated participants’ trust behavior. We expected partisan differences in trust toward Democrats and Republicans to be exacerbated when partners were more versus less threatening to perceivers.

## Method

### Participants

200 participants (*M*_age_ = 42.91, SD = 11.83) from the USA were recruited using the MTurk Toolkit (Hauser et al., 2023) from Cloud Research (Litman et al., 2017). Given the within-subject nature of the threat manipulation, we targeted a sample size of 200 participants, which is similar to related work (e.g., Malloy et al., 2023).

## Procedure

Participants read, “In this task, you will play a game with six different partners. In each game, you will be given four points. You will choose to share none, some, or all of your points with a partner. Points shared with partners are then quadrupled. Then, the other partner has the option of sharing half of the quadrupled value back with you. Or, the other partner can keep all the points. For example, if you share two points, the partner you shared with will now have eight points. That partner can choose to share four points back with you. Or, that partner can choose to keep all eight points. You will be making the initial choice about what to share with each partner before moving forward with the games.”

Participants then advanced to a second instructions screen. They read, “We will give you some information about each partner. First, we will let you know whether the partner identifies as a Republican or a Democrat. Second, we will let you know one of their responses to a questionnaire about how they think the political party with which they affiliate should function. Partners indicated whether they would like to see their party be the dominant party in American politics for the foreseeable future in creating and enacting legislation, or, whether they would like to see bipartisan efforts for the foreseeable future in creating and enacting legislation. Some partners have chosen not to identify with either party, so no additional information will be provided.”

Participants then completed the trust task, which consisted of six trials. In each trial, participants saw, “You are playing with [Partner’s name]. [Partner’s name] [identifies as a Republican/identifies as a Democrat/doesn’t identify as a Republican or Democrat]. [Partner’s name] [would like to see their party be the dominant party in American politics for the foreseeable future in creating and enacting legislation/would like to see bipartisan efforts for the foreseeable future in creating and enacting legislation/did not complete the survey about how they think their political party should function in American politics].

Below this text, participants read, “You have four points. How many points do you want to share with [Partner’s name]? Remember, after you share, the number of shared points will be quadrupled. [Partner’s name] can either share back half of the quadrupled value or keep the quadrupled value.” Participants then selected how much of their four points (0 – 4) they would like to share with the partner.

Participants did not play games with real partners. Instead, we manipulated partner political party [Democrat or Republican] and threat [less, more] within-participants in four of the six trials. The other two trials were of partners who didn’t identify as Republican or Democrat and who ostensibly did not complete the survey.

### Threat manipulation check

After the six trials, participants indicated how threatening they found people agreeing with four statements to be on a scale ranging from 1 *(not at all threatening)* to 7 *(extremely threatening)*. Participants rated how threatening they found [Republicans/Democrats] who [would like to see their party be the dominant party for the foreseeable future in creating and enacting legislation/who would like to see bipartisan efforts for the foreseeable future in creating and enacting legislation] to be. The four statements were presented in a random order.

### Participant characterization

Participants indicated their political ideology as in Study 1 (Cronbach’s *α* = .93). Participants indicated their perceived threat from Republicans (Cronbach’s *α* = .97), Democrats (Cronbach’s *α* = .96), and people having no affiliation (Cronbach’s *α* = .94) as in Study 1. Composite ideology and threat scores were created as in Study 1. See Table 1 for descriptives and intercorrelations. Participants also indicated which of the two major political parties (Republican or Democrat) they would join if they had to choose one (*N*_Republican_ = 79, *N*_Democrat_ = 121).

## Results

### Verifying manipulated threat

We first verified that the threat manipulation linked to participants’ political ideologies. That is, participants should perceive opposing versus similar partisans as more threatening in the “more threat” condition to a greater extent than in the “less threat” condition. In a multilevel model, we regressed ratings (standardized) from the threat manipulation check on Party (Republican, Democrat), Threat (less, more), participants’ composite political ideology (standardized around the overall mean) and their interactions. The random effects structure allowed for intercepts to vary by participant. It also included random slopes such that Partner Political Party effects and Threat effects were allowed to vary by participant. See Table 4 for all model coefficient information beyond the coefficient for the three-way interaction of interest that validates the threat manipulation. All effects were subsumed by the expected three-way interaction, *B* = 0.63, *p* < .001. For descriptive purposes here, we describe how more conservative and more liberal participants (defined as being one standard deviation above and below the group mean) rated threat from Democrats and Republicans in each condition.

**Table 4:** Standardized model coefficients for the threat manipulation check in Study 2

	**Threat Rating (standardized)**		
*Predictors*	*Estimates*	*CI*	*p*
(Intercept)	− 0.39	− 0.52–− 0.25	< 0.001
Participant ideology (standardized)	− 0.01	− 0.14–0.13	0.907
Political Party [Republican]	0.18	0.04–0.32	0.012
Threat [more]	0.47	0.33–0.61	< 0.001
Participant ideology (standardized) × Party [Republican]	0.14	− 0.00–0.28	0.052
Participant ideology (standardized) × Threat [more]	− 0.26	− 0.40–− 0.12	< 0.001
Party [Republican] × Threat [more]	0.26	0.10–0.42	0.001
Participant ideology (standardized) × Party [Republican] × Threat [more]	0.63	0.47–0.79	< 0.001

Conservative participants rated Democrats (Estimate = 2.87, SE = 0.20) and Republicans (Estimate = 2.96, SE = 0.19) as similarly threatening in the less threat condition. Liberal participants rated Republicans (Estimate = 3.49, SE = 0.19) as more threatening than Democrats (Estimate = 2.84, SE = 0.19) in the less threat condition. Validating the threat manipulation, these differences were exacerbated in the more threat condition. Conservative participants rated Democrats (Estimate = 4.37, SE = 0.18) as more threatening than Republicans (Estimate = 3.70, SE = 0.17) in the more threat condition. Liberal participants rated Republicans (Estimate = 5.74, SE = 0.18) as more threatening than Democrats (Estimate = 3.26, SE = 0.18) in the more threat condition.

### Threat as a moderator of partisanship effects

Our next analysis had two goals. First, we wanted to replicate the interaction between Participant Ideology and Partner Political Party shown in Study 1. Second, we wanted to test if Threat moderated this two-way interaction that reflected partisanship effects. To this end, we regressed trust (operationalized as in Study 1; standardized) on Partner Party, Threat, participants’ composite political ideology (standardized around the overall mean), and their interactions. The random effects structure allowed for intercepts to vary by participant. It also included random slopes such that Partner Party effects and Threat effects were allowed to vary by participant. We present data from the four trials in which Partner Party and Threat was fully crossed. Including the two trials with partners having no affiliation did not change nature of the findings. See Table 5 for model coefficient information beyond the below-described coefficients that were directly relevant to our hypotheses. See the Supplemental Material for results from an exploratory multilevel model using participants’ categorically defined political party instead of their continuously defined political ideology.

**Table 5:** Effects of participant ideology, partner political party, and threat on trust in Study 2. Model coefficients are standardized

	Trust (standardized)		
*Predictors*	*Estimates*	*CI*	*p*
(Intercept)	0.23	0.10–0.36	< 0.001
Participant ideology (standardized)	0.18	0.05–0.31	0.006
Partner political party [Republican]	− 0.18	− 0.32–− 0.04	0.013
Threat [more]	− 0.24	− 0.36–− 0.13	< 0.001
Participant ideology (standardized) × Partner political party [Republican]	− 0.44	− 0.58–− 0.30	< 0.001
Participant ideology (standardized) × Threat [more]	0.09	− 0.02–0.20	0.107
Partner political party [Republican] × Threat [more]	− 0.08	− 0.21–0.04	0.187
Participant ideology (standardized) × Partner Political Party [Republican] × Threat [more]	− 0.14	− 0.26–− 0.01	0.029

### Replicating study 1

As in Study 1, an interaction between Partner Party and Participant Political Ideology emerged, *B* = − 0.44. *p* < .001. We described this interaction using simple slopes like Study 1. Like Study 1, participants with more liberal ideologies trusted Republican partners less, *B* = − 0.28, SE = 0.06. Unlike Study 1, they trusted Democrat partners more, *B* = 0.23, SE = 0.06.

### Extending study 1

**Fig. 3:**
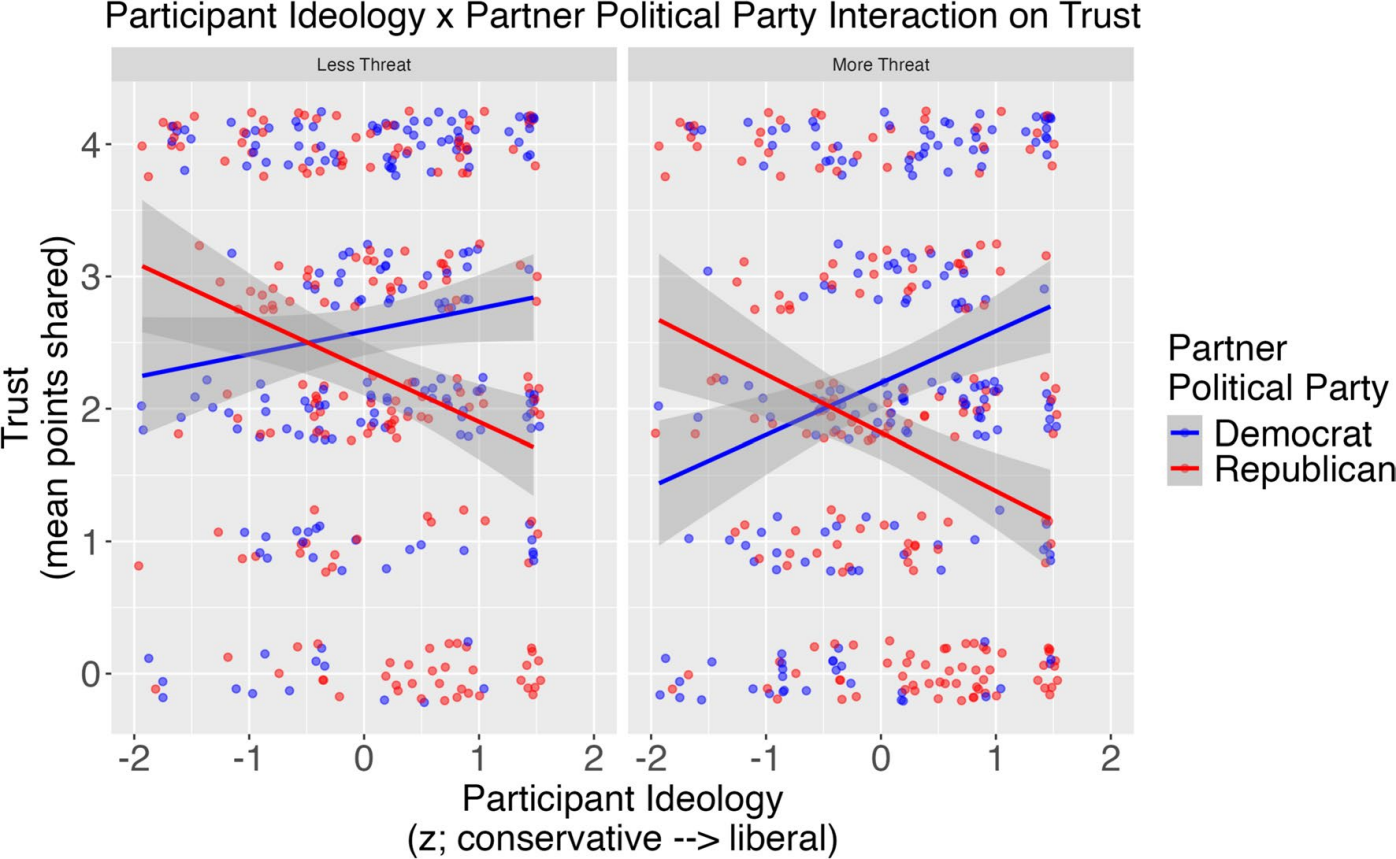
An interaction effect obtained from a multilevel model showed that participant ideology relations with trust were stronger when the partner was manipulated to be more versus less threatening in Study 2

Extending Study 1 and supporting our hypothesis, Threat qualified the interaction between Partner Political Party and Participant Political Ideology (Fig. 3), *B* = − 0.14. *p* = .03. We describe this interaction using simple slopes. In the less threatening condition, more liberal participants trusted Republican partners less, *B* = − 0.26, SE = 0.07, and Democrat partners more, *B* = 0.18, SE = 0.07. This difference appeared pronounced in the more threatening condition. Again, more liberal participants trusted Republican partners less, *B* = − 0.31, SE = 0.07, and Democrat partners more, *B* = 0.27, SE = 0.07.

## Discussion

Like Study 1, participants with more liberal ideologies trusted Republican partners less. Unlike Study 1, participants with more liberal ideologies trusted Democrat partners more. These patterns emerged despite a similar ideological profile of participants across studies. Although we can only speculate as to why these differences emerged, we offer context as potential reason. There could be differences in how college-aged participants (Study 1) and a more middle-aged sample (Study 2) approach thinking about partisans. It could also be that the real national political environment in which a study is conducted affects trust behavior. For example, participants’ ideology in Study 1 did not significantly relate to the threat they perceived from Democrats. By contrast, more liberal ideologies negatively related to perceived threat from Democrats in Study 2. It could be that being a college environment replete with a variety of ideologies reduces the threat perceived from at least some partisans. It could also be that conducting a study when different political parties are in power nationally (e.g., Republicans in 2025) affects the nature of participants’ responses. These possibilities align with work suggesting that partisanship effects are best assessed in the contexts of different social settings in which people find themselves (Klar, 2014).

Study 2 provided a critical extension of Study 1 by experimentally manipulating perceived partisan threat. This manipulation allowed us to determine whether perceived threat might be a mechanism by which people make trust decisions about other partisans. Surprisingly little work (but see Malloy et al., 2023) has manipulated threat to evince such a mechanism. We find support for threat as a mechanism for partisan trust decisions. Although participants always trusted similar versus opposing partisans more, this pattern was exacerbated in the more versus less threatening condition.

### General discussion

Across two studies, we provide evidence of interactive perceiver and target partisanship effects on simple decisions reflecting trust. Across studies, the data support the idea that people trust opposing (versus similar) partisans less. Such findings are consistent with affective polarization (Iyengar et al., 2019) that negatively impacts complex social interactions in both partisan and non-partisan contexts (Mamakos & Finkel, 2023). The current work builds on such work by showing that such polarization is also present in simple decisions that require trust.

Consistent with work suggesting affective polarization to be driven by threat (Renström et al., 2021), Study 1 provided correlational evidence that the extent to which an oppositional group is affect-laden relates to trust behavior. Study 1 additionally suggested that people’s political ideologies may relate to partisan trust decisions via their association with threat (at least threat from Republicans). These findings are consistent with a socially sectarian view of American politics (Finkel et al., 2020). Speculatively, rather than an aspect of the self (e.g., ideology) consistently relating to trust decisions toward partisans, perhaps perceptions of the *othered* group (e.g., the threat they pose) more consistently relate to these decisions. Aligning with this idea, Study 2 provided causal evidence that partisan threat affects the relation between people’s political leanings and partisan trust decisions. Together, these findings provide more evidence for affective and likely threat-driven polarization shaping the social landscape in which we live (e.g., Abramowitz & Webster, 2016).

The threat-based account of affective polarization supported in trust behavior here has many implications. For example, partisans who are more likely to exhibit cross-partisan animus differentially respond to public health messaging from partisan leaders (Druckman et al., 2020) to the extent that it affects their health decisions (Gollwitzer et al., 2020). The current findings allow for the possibility that trust decisions rooted in affective polarization do not only emerge for how people respond to messages from leaders. They also emerge in the simple everyday decisions to trust that have ramifications for who forms the intergroup relations known to reduce prejudice (Pettigrew, 1998). Because contact with opposing partisans can reduce affective polarization (e.g., Whitt et al., 2021), one potentially fruitful area for research could be to examine whether the extent of inter-partisan contact or isolation affects behavioral trust decisions in various contexts.

It is also important to acknowledge trust as a learned behavior (King-Casas et al., 2005). Conducting analyses on data from an integrative trust game would allow for the opportunity to learn from feedback about trust behavior instead of relying solely on initial perceptions. As participants interacted with each partner only once (in the analyzed data for Study 1), there is no information on learning over repeated interactions. That the findings from Study 1 and Study 2 are largely consistent, however, suggests that the analyzed data in Study 1 coming from the first trial of a multi-trial game did not affect behavior.

Yet, this possibility does not mean that people’s subsequent decisions in a multilevel trust game would simply mirror what happens in a first encounter. People may approach a first encounter differently than they would an encounter after several previous interactions, potentially affecting their decision making and trust behavior. This type of design would not only allow for investigating the influence of group membership perceptions but also how trust behavior from the partners (some being less trustworthy than others by sharing points back less often) impacts learning as a function of group membership. Furthermore, in real-world scenarios, trust is often dynamic and can evolve based on ongoing interactions and feedback. Using an iterative task would also allow learning of trust information in the context of perceived threat to be studied. In much the same way that learning may take place at a different rate due to group membership, it could also be significantly impacted by levels of perceived threat. By implementing an iterative trust game, researchers could capture the learning process and observe how trust behavior changes over time as participants gain more information about the partner’s trustworthiness in social contexts. Such research is important considering that computational modeling can offer precise accounts for mechanisms governing social behavior (Hackel & Amodio, 2018).

The current work adds to growing evidence that partisanship clouds everyday social decisions (Cassidy et al., 2022; Mallinas et al., 2018; Malloy et al., 2023; Nicholson et al., 2016) by revealing its effects on trust behaviors. By identifying potentially linked effects of participant political ideology and perceived partisan threat on behavioral trust, we highlight an important role of both perceiver and target characteristics that may contribute to partisan tension not only toward leaders, but to everyday people belonging to political coalitions. These effects on trust behaviors are likely essential to address when considering the development of interventions aimed at improving cross-party interactions in the general voting population.

## Supplementary Information

Supplementary file 1.

## Data Availability

All data and analytic code are available at https://osf.io/5b4gs/?view_only=b6a9c9b13e734288be8c946b6fcc947b
