# Supplementary material for: Political partisanship and perceived partisan threat relate to simple trust decisions
Source: Cogn Res Princ Implic. 2026 Jan 3;11:1. doi: 10.1186/s41235-025-00698-3 (PMC12764723; doi:10.1186/s41235-025-00698-3)
Supplement: Supplementary file 1 [file 41235_2025_698_MOESM1_ESM.docx]

**Supplemental Material**

**Supplemental Table 1**

*Effects of participant political ideology and partner political party on trust (controlling for the amount earned in the first trial with a same-party player) in Study 1*

|  | **Amount Shared with Second Player** | | |
| --- | --- | --- | --- |
| *Predictors* | *Estimates* | *CI* | *p* |
| (Intercept) | 1.79 | 1.64 – 1.95 | <0.001 |
| Participant Ideology (standardized) | -0.20 | -0.38 – -0.03 | 0.023 |
| Partner Political Party [Republican] | -0.20 | -0.33 – -0.06 | 0.004 |
| **Amount earned in last encounter with the party**  **(standardized)** | **0.23** | **0.07 – 0.38** | **0.004** |
| **Participant Ideology × Partner Political Party**  **[Republican]** | **-0.17** | **-0.33 – -0.02** | **0.023** |
| Participant Ideology ×  Amount earned in last encounter with the party | 0.08 | -0.09 – 0.25 | 0.345 |
| Partner Political Party [Republican] × Amount earned in last encounter with the party | 0.03 | -0.12 – 0.18 | 0.732 |
| Participant Ideology ×  Partner Political Party [Republican] × Amount earned in last encounter with the party | -0.10 | -0.27 – 0.07 | 0.240 |
|  | | | |
|  |  | | |
|  |  | | |
|  |  | | |
|  |  | | |

**Study 2 Analyses using Categorically-Defined Partisanship**

Study 2 extended Study 1 by additionally analyzing the data based on whether participants identified with Republicans or Democrats. Past research suggests that continuously measured political ideology is sufficient to capture partisan differences in person perception (e.g., Wilson & Rule, 2014). However, if a participant sample is more ideologically liberal, for example, labeling participants one standard deviation below a more liberal mean may not elicit patterns reflecting a more conservative viewpoint as well as Republican self-identifications. We expected the findings using participant political affiliation to parallel the patterns found when using continuously measured political ideology. The Study 2 sample had a similar ideological profile as Study 1 sample. That more participants were ideologically liberal means that a participant one standard deviation below the mean composite ideology score might not best reflect conservative viewpoints and affiliations. Indeed, more participants identified as Democrat than Republican. The threat manipulation may affect trust more when analyzing participants by party affiliations versus ideology.

We thus re-ran the Study 2 analyses but included participants’ political party (i.e., Republican or Democrat) instead of continuously measured political ideology in the model. See Supplemental Table 2 for coefficient information. The interaction between Partner Political Party and Participant Party was significant. Democrat (*Estimate* = 2.64, *SE* = 0.11) versus Republican (*Estimate* = 1.96, *SE* = 0.13) participants trusted Democrat partners more. Democrat (*Estimate* = 1.62, *SE* = 0.11) versus Republican (*Estimate* = 2.73 *SE* = 0.14) participants trusted Republican players less.

These effects were qualified by the three-way interaction. We describe this interaction by comparing trust behavior toward Democrat and Republican players between Democrat and Republican participants at each level of threat. In the less threatening condition, Democrat (*Estimate* = 2.76, *SE* = 0.12) versus Republican (*Estimate* = 1.93, *SE* = 0.12) participants trusted Democrat players more. Democrat (*Estimate* = 2.22, *SE* = 0.15) versus Republican (*Estimate* = 2.84, *SE* = 0.15) participants trusted Republican players less. These patterns appeared pronounced. Democrat (*Estimate* = 2.51, *SE* = 0.12) versus Republican (*Estimate* = 1.30, *SE* = 0.12) participants trusted Democrat players more. Democrat (*Estimate* = 1.71, *SE* = 0.15) versus Republican (*Estimate* = 2.62, *SE* = 0.15) participants trusted Republican players less.

**Supplemental Table 2**

*Effects of participant political party and player political party on trust in Study 2*

|  | **Trust** | | |
| --- | --- | --- | --- |
| *Predictors* | *Estimates* | *CI* | *p* |
| (Intercept) | 2.76 | 2.52 – 3.00 | **<0.001** |
| Participant Political Party [Republican] | -0.55 | -0.92 – -0.17 | **0.004** |
| Partner Political Party [Republican] | -0.83 | -1.09 – -0.57 | **<0.001** |
| Threat [more] | -0.25 | -0.45 – -0.04 | **0.017** |
| Participant Political Party [Republican] ×  Partner Political Party [Republican] | 1.45 | 1.03 – 1.86 | **<0.001** |
| Participant Political Party [Republican] ×  Threat [more] | -0.26 | -0.58 – 0.07 | 0.119 |
| Partner Political Party [Republican] ×  Threat [more] | -0.39 | -0.68 – -0.10 | **0.008** |
| Participant Political Party [Republican] ×  Partner Political Party [Republican] ×  Threat [more] | 0.68 | 0.22 – 1.14 | **0.004** |
